# First record of the *Ligia baudiniana *species complex in the American Gulf of Mexico Coastline, as confirmed by morphological and molecular approaches

**DOI:** 10.12688/f1000research.12459.1

**Published:** 2017-08-30

**Authors:** Carlos A. Santamaria, Edgar T. Bischoff III, Moe Aye, Keith W. Phillips, Victoria Overmeyer

**Affiliations:** 1 Biology Program, College of Science and Mathematics, University of South Florida Sarasota-Manatee, Sarasota, FL, 34243, USA

**Keywords:** Isopoda, cryptic species, Sarasota, Crustacea, Ligia exotica

## Abstract

*Ligia* isopods exhibit a constrained morphology that makes identification difficult. In the Greater Caribbean, a convoluted taxonomic history has left the distributional limits of *Ligia baudiniana* unclear. To date, no confirmed records of this species exist from the American Gulf of Mexico. Herein, we report the presence of *L. baudiniana* in Sarasota-Manatee Florida, as confirmed by morphological and molecular approaches. This is the first record of this species in the region and a ~300Km extension of its range. Specimens were collected in mangroves, underscoring the importance of protecting these habitats.

## Introduction

The isopod genus *Ligia* includes ~40 nominal species, most of which inhabit a narrow range in the upper rocky intertidal habitats. In the Greater Caribbean Region (i.e. the Caribbean and adjacent regions), a single endemic species is currently considered valid: *Ligia baudiniana* ^1, 2^. The species has been reported from Brazil ^3^, the Caribbean islands ^4^, the Pacific coastlines of Central and South America ^5– 7^, Bermuda ^8^, Bahamas ^4^, and in southern Florida ^9, 10^ and the Everglades ^4^; however, doubt over historical records have left the distributional limits of *L. baudiniana* unclear.

*L. baudiniana* was described from specimens collected in the San Juan de Ulua Fort in Veracruz, Mexico. Milne-Edwards’ original species description ^11^ focuses on characters that are of limited taxonomic importance ^12^, lacks illustrations, and does not provide an account of male reproductive structures now known to be useful in *Ligia* taxonomy ^12– 14^. Indeed, the terse description and source origin of the type material (i.e., artificial substrate) have led to confusion on whether *L. baudiniana* is a synonym of *L. exotica* or a valid species ^15, 16^, and to records and specimens identified as *L. baudiniana* to be re-classified as *L. exotica* ( 3 and references). This is particularly true for specimens from the American Gulf of Mexico coastlines, as most records appear to have been reclassified as *L. exotica*. Furthermore, a wide-ranging survey of *Ligia* in the Gulf of Mexico from Texas to Florida has shown artificial habitats in the region to harbor only *L. exotica* (unpublished study; Hurtado LA, Mateos M, Wang C, Santamaria CA, Jung J, Khalaji-Pirbalouty V, and Kim W).

**Figure 1.**
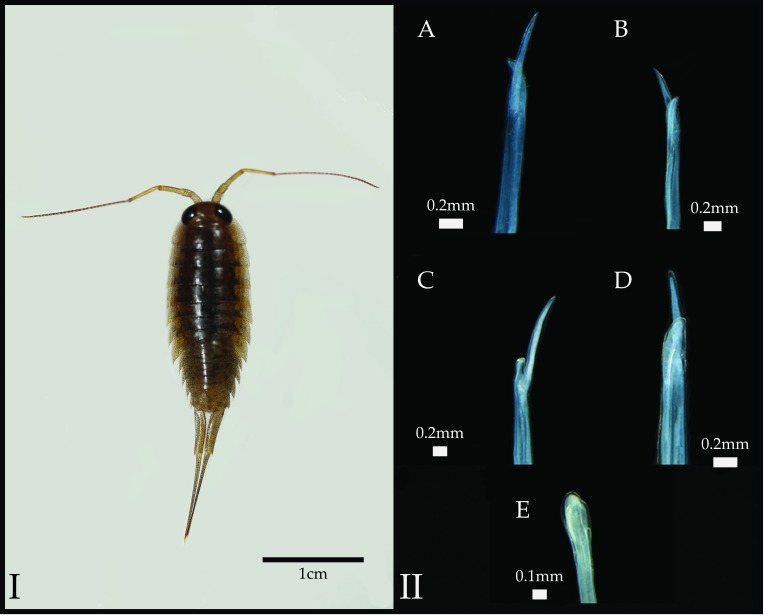
*Ligia baudiniana* (I) and its characteristic appendix masculina (II **A**– **D**). This trait was used to putatively identify specimens collected in this study. The appendix masculine of *L. exotica* is shown in II-E. Photographs in panel II are reproduced under a Creative Commons license from Santamaria *et al*. (2014) ^14^.

The taxonomic confusion between *L. baudiniana* and *L. exotica* is complicated by the presence of a *Ligia* species endemic to habitats throughout the Greater Caribbean, Gulf of Mexico excluded, that is easily recognized by a unique male gonopod morphology that is readily distinguishable from *L. exotica* ( Figure 1), and that has been attributed to *L. baudiniana* by Andersson ^5^, Rouse ^9^, Schultz ^4, 8^, and Schultz and Johnson ^10^. A recent molecular study demonstrated that *Ligia* exhibiting this trait form a well-supported monophyletic clade composed of several cryptic and highly divergent lineages endemic to the region ^14^. The combination of these studies suggests that *L. baudiniana* as currently recognized: (a) is an endemic species to the Greater Caribbean Region; (b) can be identified using both molecular and morphological tools; and (c) appears to have a broad geographic range that includes the Caribbean islands, the Pacific coastlines of Central America to Ecuador, Bermuda, Bahamas, and southern Florida.

In southern Florida, *L. baudiniana* is reported from the Florida Keys ^9, 10^ and the Everglades ^4^, while no confirmed records from the American Gulf of Mexico exist to date. In this study, we use molecular and morphological approaches to identify specimens collected from Sarasota and Manatee counties in Florida as *L. baudiniana*. Our findings extend the confirmed range of this species ~300-km into the Gulf of Mexico coastline of Florida and represent the first confirmed record of *L. baudiniana* in the American Gulf of Mexico coastline.

## Methods

**Figure 2.**
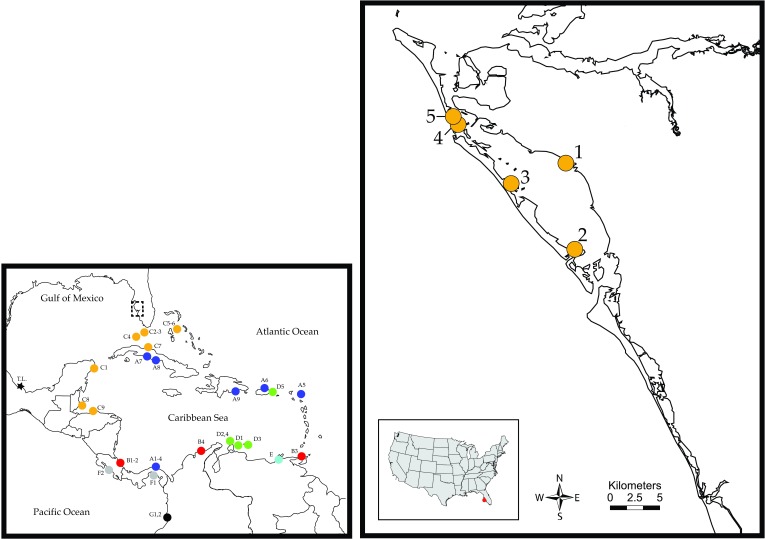
Locations sampled in Sarasota and Manatee counties, Florida. Locations are: (SRQ1) End of Tiara Drive; (SRQ2) Quick Point; (SRQ3) Joan M. Durante Community Park; (SRQ4) Leffis Key; (SRQ5) Near Coquina Beach. Detailed locality information can be found in Table 1. The smaller panel presents the distribution of *L. baudiniana* lineages reported to date throughout the Caribbean and its adjacent region.

*Ligia* specimens were collected by hand across the Sarasota-Manatee counties in Florida ( Table 1, Figure 2) and field preserved in 70% EtOH. No permits were necessary for collections. Specimens were identified to species by inspecting the morphology of the apex of the endopod of the second pleopod of 15–25 male *Ligia* specimens per site, with individuals putatively identified as *L. baudiniana* if they exhibited a large process bifurcating close to the apex of the appendix masculina ( Figure 1A), as proposed by Schultz ^4, 8^ and confirmed by Santamaria *et al.* ^14^. A subset of specimens was deposited in the Invertebrate Collections of the Biodiversity Research and Teaching Collections (BRTC) at Texas A&M University ( http://brtc.tamu.edu/).

**Table 1.** Localities included in analyses and corresponding species ID, geographic information, GenBank accession numbers, and BRTC voucher numbers when applicable. New records are in **bold**.

Species	Locality	Label or haplotype name	Latitude	Longitude	GenBank Accession No.	Museum Voucher
***L. baudiniana***	**End of** **Tiara Drive,** **Bradenton, FL,** **U.S.A.**	**SRQ1**	**27°24'45.48"N**	**82°34'56.60"W**	**MF668214** **MF668218**	**TCWC 2-4741**
***L. baudiniana***	**Quick Point,** **Longboat Key,** **FL, U.S.A.**	**SRQ2**	**27°20'19.10"N**	**82°34'56.49"W**	**MF668216** **MF668219** **MF668220** **MF668223**	**TCWC 2-4737**
***L. baudiniana***	**Joan M Durante** **Community** **Park, Longboat** **Key, FL, U.S.A.**	**SRQ3**	**27°24'56.40"N**	**82°39'19.65"W**	**MF668217** **MF668222** **MF668225**	**TCWC 2-4740**
***L. baudiniana***	**Leffis Key,** **Bradenton** **Beach, FL, U.S.A.**	**SRQ4**	**27°27'08.64"N**	**82°41'17.25"W**	**MF668224**	**TCWC 2-4739**
***L. baudiniana***	**Gulf Drive** **South,** **Bradenton** **Beach, FL, U.S.A.**	**SRQ5**	**27°27'21.07"N**	**82°41'36.37"W**	**MF668215** **MF668221** **MF668224** **MF668226**	**TCWC 2-4738**
*L. baudiniana*	Cozumel, Mexico	C1_2	20°25'13.64"N	86°50'42.26"W	KF555855	N/A
*L. baudiniana*	Indian Key, FL, U.S.A.	C3_2	24°53'23.70"N	80°40'31.38"W	KF555859	N/A
*L. baudiniana*	Summerland Key, FL, U.S.A.	C4_1	24°39'07.62"N	81°26'09.48"W	KF555860	N/A
*L. baudiniana*	Nassau, The Bahamas	C5_1	25°04'47.22"N	77°22'11.52"W	KF555858	N/A
*L. baudiniana*	Jaws Beach, The Bahamas	C6_1	25°01'05.05"N	77°32'49.00"W	KF555862	N/A
*L. baudiniana*	Habana, Cuba	C7_2	N/A	N/A	KF555861	N/A
*L. baudiniana*	Long Bird Bridge, Bermuda	C10_4	32°21'05.34"N	64°42'35.16"W	KF555856	N/A
*L. baudiniana*	Stonehole Bay, Bermuda	C12_1	32°15'19.62"N	64°48'49.68"W	KF555857	N/A
*L. baudiniana*	Fort Sherman, Panama	A1_1	09°21'51.36"N	79°56'55.56"W	KF555844	N/A
*L. baudiniana*	Portobelo (B), Panama	A2_1	09°32'14.72"N	79°40'26.30"W	KF555843	N/A
*L. baudiniana*	Portobelo (C), Panama	A3_1	09°32'54.24"N	79°40'14.10"W	KF555846	N/A
*L. baudiniana*	Portobelo (A), Panama	A4_1	09°33'11.70"N	79°39'35.58"W	KF555845	N/A
*L. baudiniana*	Yaguanabo, Cuba	A7_2	N/A	N/A	KF555849	N/A
*L. baudiniana*	Playa Ancon, Cuba	A8_1	N/A	N/A	KF555848	N/A
*L. baudiniana*	Boca Chica, Dominican Republic	A9_1	18°26'37.02"N	69°36'37.98"W	KF555847	N/A
*L. baudiniana*	Playa Bonita, Costa Rica	B1_1	10°00'39.59"N	83°03'46.87"W	KF555850	N/A
*L. baudiniana*	Piuta, Costa Rica	B2_1	10°00'20.70"N	83°02'06.92"W	KF555851	N/A
*L. baudiniana*	Santa Marta, Colombia	B4_2	11°20'07.74"N	73°58'31.26"W	KF555852	N/A
*L. baudiniana*	Piscaderabaai, Curacao	D1_1	12°07'25.38"N	68°58'09.30"W	KF555866	N/A
*L. baudiniana*	Spaans Lagoen, Aruba	D2_1	12°27'45.18"N	69°58'00.42"W	KF555865	N/A
*L. baudiniana*	Donkey Beach, Bonaire	D3_1	12°07'50.10"N	68°17'04.44"W	KF555867	N/A
*L. baudiniana*	East Coast, Aruba	D4_1	12°32'44.58"N	69°57'46.68"W	KF555868	N/A
*L. baudiniana*	Fajardo, Puerto Rico	D5_1	18°21'38.84"N	65°37'28.51"W	KF555869	N/A
*L. baudiniana*	Veracruz, Panama	E1_1	08°53'28.30"N	79°35'35.19"W	KF555863	N/A
*L. baudiniana*	Caldera, Costa Rica	E2_1	09°56'26.96"N	84°44'02.93"W	KF555864	N/A
*L. baudiniana*	Buenaventura, I. Palma, Colombia	G1_1	N/A	N/A	KF555871	N/A
*L. baudiniana*	Maguipi, Colombia	G1_2	N/A	N/A	KF555870	N/A
*L. baudiniana*	Buenaventura, I. Palma, Colombia	G2_1	N/A	N/A	KF555872	N/A
*L. exotica*	Multiple localities in China	CH12	N/A	N/A	JX414150	N/A
*L. exotica*	Multiple localities in China	CH13	N/A	N/A	JX414151	N/A
*L. exotica*	Multiple localities in China	CH14	N/A	N/A	JX414152	N/A
*L. exotica*	Multiple localities in China	CH15	N/A	N/A	JX414153	N/A
*L. exotica*	Multiple localities in China	CH16	N/A	N/A	JX414154	N/A
*L. exotica*	Multiple localities in China	CH17	N/A	N/A	JX414155	N/A
*L. exotica*	Multiple localities in China	CH18	N/A	N/A	JX414156	N/A
*L. exotica*	Multiple localities in China	CH19	N/A	N/A	JX414157	N/A
*L. exotica*	Multiple localities in China	CH20	N/A	N/A	JX414158	N/A
*L. exotica*	Multiple localities in China	CH21	N/A	N/A	JX414159	N/A
*L. exotica*	Multiple localities in China	CH22	N/A	N/A	JX414160	N/A
*L. exotica*	Fort Johnson, Charleston, South Carolina, USA	Ligia exotica	N/A	N/A	GU270929	N/A
*L. exotica*	Veracruz Harbor	Out_CAR30_1	19°11'40.19"N	96°07'24.41"W	KF546664	N/A
*L. exotica*	Indian Fields Creek, Virginia, USA	SERCINVERT0370	37°16'04.80"N	76°33'21.69"W	KU906047	N/A

Morphological identifications were corroborated using a mitochondrial barcoding approach. We extracted total genomic DNA from pleopods/pereopods for a subset of individuals putatively identified as *L. baudiniana* using the ZR Quick-gDNA Miniprep Kit. Previously described primers and conditions were used to PCR-amplify and sequence a 658-bp fragment of the Cytochrome Oxidase I gene (COI, primers LCO1490/HCO2198; 17). Positive amplicons were cleaned and sequenced at the University of Arizona Genetics Core (UAGC). Sequences were assembled in Geneious R v8.1.7.

We combined nucleotide sequences produced in this study with publicly available ones for *L. baudiniana* and *L. exotica* ( Table 1). We used default settings to align the resulting dataset using the MUSCLE Alignment ^18^ tool in Geneious R v8.1.7. No signs of misaligned regions or pseudo-genes were observed in the resulting alignment. The final alignment was imported into MEGA v7.0.18 ^19^, where we estimated a neighbor-joining tree under Kimura’s 2-parameter model (hereafter K2P; 20) and uniform rates. Support for the relationships within the tree were estimated by conducting 1,000 bootstrap replicates. Lastly, we calculated K2P genetic distances between haplotypes produced by this study, *L. exotica*, and previously reported *L. baudiniana* clades ^14^.

## Results

**Figure 3.**
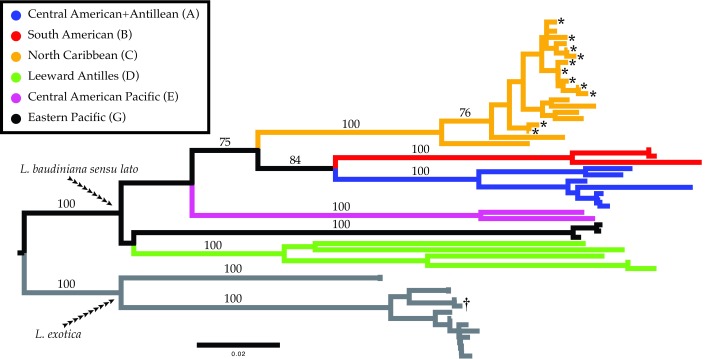
Neighbor-Joining phylogram of COI haplotypes for *Ligia baudiniana* and *L. exotica*. Molecular identifications of putative *L. baudiniana* samples from Sarasota were made using K2P distances. All haplotypes for *Ligia* from Sarasota-Manatee counties (denoted by an *) are placed with previously reported haplotypes from the North Caribbean Clade reported by Santamaria *et al.* ^14^ in a well-supported clade (values near nodes represent bootstrap support values). Branches are drawn to scale, with colors and labels corresponding with those used by Santamaria *et al.* ^14^. The COI haplotype obtained from topotypes of *L. baudiniana* by Santamaria *et al.* ^14^ is denoted by a †.

Molecular identifications produced results congruent with morphological identifications. We obtained 12 unique COI haplotypes from a total of 25 individuals putatively identified as *L. baudiniana.* Haplotypes produced in this study were highly similar to each other (COI K2P 0.00–2.81%, Table 2) and to those reported from localities in the Florida Keys, The Bahamas, northern Cuba, Cozumel, and Bermuda (COI K2P 0.50–6.08%, Table 2). Haplotypes were moderately to highly divergent from *L. baudiniana* from other localities in the Caribbean (COI K2P 14.44–24.90%, Table 2), and highly divergent from *L. exotica* (COI K2P 20.32–25.18%). The neighbor-joining analysis produced similar results ( Figure 3), nesting all haplotypes produced in this study in a well-supported clade (Bootstrap Support = 100) with the *Clade C* reported by Santamaria *et al.* ^14^. All unique haplotypes have been deposited in GenBank ( Table 1).

**Table 2.** Divergence estimates between and within *Ligia baudiniana* lineages as estimated by K2P distances. The top diagonals show minimum and maximum divergences between lineages, with lower diagonals presenting average genetic distances between clades. Within-group divergences are shown in the middle diagonal (in bold) in the order: minimum, maximum, and average divergence.

	Sarasota- Manatee (SRQ)	North Caribbean (C)	Central American + Antillean (A)	South American (B)	Leeward Antilles (D)	Central American Pacific (E)	Eastern Pacific (G)	*L. exotica*
Sarasota- Manatee (SRQ)	**0.00-2.80** **1.20**	0.50-18.0	14.4-18.9	15.4-19.0	21.3-24.9	17.5-19.3	21.1-23.6	20.3-25.2
North Caribbean (C)	5.50	**0.30-19.1** **8.20**	13.6-18.9	13.8-19.3	18.7-25.6	17.2-21.7	20.7-23.5	20.7-27.0
Central American + Antillean (A)	16.2	16.0	**0.30-7.80** **4.20**	13.8-16.2	20.0-23.7	20.0-23.7	17.8-21.6	23.5-27.1
South American (B)	17.4	17.2	15.0	**0.30-5.20** **3.50**	20.2-25.2	19.6-22.8	22.2-23.9	23.7-30.0
Leeward Antilles (D)	23.1	22.6	21.4	21.7	**0.80-17.0** **12.8**	19.1-23.6	21.0-23.9	22.4-27.8
Central American Pacific (E)	18.5	18.9	21.7	21.2	21.0	**N/A-N/A** **5.20**	21.1-23.2	21.8-27.9
Eastern Pacific (G)	22.3	22.2	19.7	22.8	22.7	22.3	**0.30-1.00** **0.70**	22.5-24.1
*L. exotica*	23.4	23.7	25.2	25.0	25.2	25.5	23.2	**0.00-14.9** **5.10**

## Discussion

Morphological and molecular evidence confirm that our sampled individuals represent *L. baudiniana*. These new records represent the first confirmed presence of this species in the Gulf of Mexico coastlines of the USA and extend the recognized range of the species ~300 km northward from a previous confirmed record from Florida Bay. Positive identifications in this study were made using both morphological and molecular characters. These findings are important as Florida’s rich coastal biodiversity faces serious threats such as sea-level rise, introduction of alien species, urbanization, habitat loss, and species displacements ^21^.

All *L. baudiniana* specimens collected in our surveys were found in coastal mangrove forests with no specimens found in >10 surveyed artificial habitats. This suggests that coastal development in the American Gulf of Mexico may have led to the replacement of a native species with an introduced one via the removal of mangrove habitats for the establishment of artificial substrates. Additional work is needed to establish whether *L. baudiniana* is present in other mangrove habitats along the Gulf of Mexico, thus clarifying the northern limits of this species’ range.

## Supplementary material

Supplementary File 1: Alignment of COI gene sequences for all sequenced individuals and *L. baudiniana* and *L. exotica* sequences from GenBank.
