# Supplementary material for: First record of the Ligia baudiniana species complex in the American Gulf of Mexico Coastline, as confirmed by morphological and molecular approaches
Source: F1000Res. 2017 Aug 30;6:1602. [Version 1] doi: 10.12688/f1000research.12459.1 (PMC5691373; doi:10.12688/f1000research.12459.1)
Supplement: Peer review history

# Peer review: Referee response for version 1

**DOI:** 10.5256/f1000research.13491.r25492

**Published:** 2017-11-16

**Authors:** Stefano Taiti

**Affiliations:** 1 Institute of Ecosystem Studies (ISE), National Research Council (Italy), Sesto Fiorentino, Italy

This is a very interesting paper  which can provide clear differences between two species of *Ligia* which have been mixed up in papers published up to the middle of last century.

 I agree that the main differential character between *L. baudiniana* and *L. exotica* is the shape of male pleopod 2 endopod, even if several other morphological characters differ between the two species (e.g. shape of telson). In the text and Figure 1 the male pleopod 1 endopod is called “appendix masculina” or “gonopod”. I would avoid using these terms, since they are not in use in the taxonomy of terrestrial isopods, also because they might not refer to the same appendage. In *Ligia* the male modifications are present mainly in the pleopod 2 endopod, but in other genera of Oniscidea they are present on the pleopod 1 endopod or even on both pleopod 1 and 2 endopods.

*Ligia baudiniana* is reported also from the Galapagos.

I have read this submission. I believe that I have an appropriate level of expertise to confirm that it is of an acceptable scientific standard.

# Peer review: Referee response for version 1

**DOI:** 10.5256/f1000research.13491.r26865

**Published:** 2017-10-11

**Authors:** Mary K. Wicksten

**Affiliations:** 1 Texas A&M University, College Station, TX, USA

Very nice work, well supported with clear diagrams and photographs. Double-check spelling: Introduction line 4: doubt has or doubts have...I wonder if further work will show that the reports from the eastern Pacific constitute a sibling species but this information is not pertinent to acceptance of the current manuscript. It might be worthwhile to point out that mangroves either do not occur or do not support characteristic communities in the northern Gulf of Mexico.

I have read this submission. I believe that I have an appropriate level of expertise to confirm that it is of an acceptable scientific standard.
